# Docking-based virtual screening of TβR1 inhibitors: evaluation of pose prediction and scoring functions

**DOI:** 10.1186/s13065-020-00704-3

**Published:** 2020-08-14

**Authors:** Shuai Wang, Jun-Hao Jiang, Ruo-Yu Li, Ping Deng

**Affiliations:** College of Pharmacy, Chongqing Medical University, Chongqing, 400016 China

**Keywords:** Docking methods, Scoring functions, Molecular docking-based virtual screening, Transforming growth factor β, Inhibitor

## Abstract

To improve the reliability of virtual screening for transforming growth factor-beta type 1 receptor (TβR1) inhibitors, 2 docking methods and 11 scoring functions in Discovery Studio software were evaluated and validated in this study. LibDock and CDOCKER protocols were performed on a test set of 24 TβR1 protein–ligand complexes. Based on the root-mean-square deviation (RMSD) values (in Å) between the docking poses and co-crystal conformations, the CDOCKER protocol can be efficiently applied to obtain more accurate dockings in medium-size virtual screening experiments of TβR1, with a successful docking rate of 95%. A dataset including 281 known active and 8677 inactive ligands was used to determine the best scoring function. The receiver operating characteristic (ROC) curves were used to compare the performance of scoring functions in attributing best scores to active than inactive ligands. The results show that Ludi 1, PMF, Ludi 2, Ludi 3, PMF04, PLP1, PLP2, LigScore2, Jain and LigScore1 are better scoring functions than the random distribution model, with AUC of 0.864, 0.856, 0.842, 0.812, 0.776, 0.774, 0.769, 0.762, 0.697 and 0.660, respectively. Based on the pairwise comparison of ROC curves, Ludi 1 and PMF were chosen as the best scoring functions for virtual screening of TβR1 inhibitors. Further enrichment factors (EF) analysis also supports PMF and Ludi 1 as the top two scoring functions.

## Background

Due to the vast improvement of computing power and the rapid development of computational chemistry and biology, computer-aided drug design (CADD) technology has been successfully applied in drug discovery, accelerating the research process and reducing the associated costs and risks [1]. Virtual screening is a common CADD technology used for the identification of hit molecules from large-scale compound libraries, providing a highly efficient approach to discover new compounds in the early stage of drug development [2]. Virtual screening methods, including molecular docking [3], pharmacophore model [4], and similarity searching [5], are widely used in drug research and development. With an increasingly large number of target protein structures being resolved, the molecular docking-based virtual screening (DBVS), has become one of the most common virtual screening methods, having achieved a great success [6].

However, due to its low accuracy, DBVS suffers from constant criticism. This method involves two basic processes: positioning ligands into a protein active site, scoring and ranking docked ligands. Both steps are imperfect. In the last decade, several studies on docking and scoring methods demonstrated that most of the docking methods and scoring functions are sensitive to the receptor, and conclusions on different receptors may be inconsistent with each other [7–10]. Different docking programs may show different performances in the same target protein [8]. There is often a poor correlation between the scores given by scoring functions and experimental binding affinities. New forms of examination are necessary to maximize the success rate of docking and scoring methods, to determine the best parameters for individual protein targets.

Transforming growth factor-β (TGFβ) signaling plays a key role in cell growth, migration, differentiation, epithelial–mesenchymal transition (EMT), and extracellular matrix remodeling [11]. TGFβ is reported to be critical in the later stages of tumorigenesis by increasing immunosuppressive Treg cells and facilitating EMT [12]. TGFβ type 1 receptor (TβR1) is a key member of the TGFβ signaling pathways, and its inhibition has the potential to enhance the immune response against tumors. Thus, TβR1 has attracted wide attention, as a potential drug target [13].

Different virtual screening models can be complementary in the search for new inhibitors. In our previous studies, several inhibitors were screened and designed by the pharmacophore model and structure–activity relationship study [14, 15]. In this study, we aimed to obtain new TβR1 inhibitors using DBVS. For that, we assessed different docking methods and scoring functions for TβR1, before large-scale database screening. We conclude that the CDOCKER protocol with a successful docking rate of 88% and the PMF and Ludi 1 scoring functions with high reliability may be efficiently applied in the virtual screening of new inhibitors of TβR1.

## Materials and methods

### Database preparation

The Protein Data Bank (PDB; http://www.rcsb.org) funded by the National Science Foundation, the National Institutes of Health, and the US Department of Energy is the single worldwide archive of 3D structural data of biological macromolecules, such as proteins, nucleic acids, and complex assemblies [16]. Up to now, 22 crystal structures of TβR1 with various small molecule inhibitors have been reported in PDB, including 1RW8, 1PY5 [17], 1VJY [18], 2WOU, 2WOT [19], 2X7O [20], 3GXL, 3HMM [21], 3FAA [22], 3KCF [23], 3TZM [24], 4X2F, 4X2G, 4X2J [25], 5E8W, 5E8Z [26], 5FRI [27], 5QIK, 5QIL, 5QIM [28], 5USQ [29], and 6B8Y (Additional file 1: Table S1) [12]. Water molecules and co-crystallized ligands were deleted and then the receptor protein was subsequently prepared by the Prepare Protein protocol using the Discovery Studio 2017R2 software package (DS; https://www.3ds.com/). The following tasks were performed: insertion of missing atoms in incomplete residues, modeling of missing loop regions, deleting of alternate conformations (disorder), standardizing of atom names, and protonating of titratable residues.

**Fig. 1:**
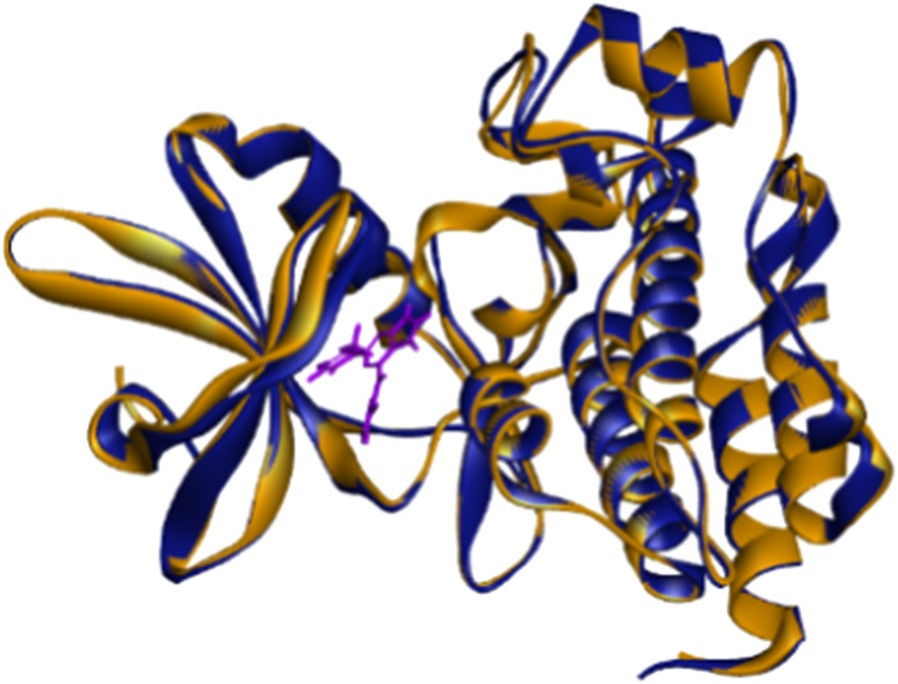
Overlay of crystal structures between 4X2F (gold ribbon) and 6B8Y (blue ribbon). The ligand in 6B8Y was shown with the purple stick model

22 X-ray structures of TβR1 deposited in the Protein Data Bank (PDB) were analyzed using some parameters to select the most suitable TβR1 structure for next studies, such as information on the resolution and organisms of crystallographic structures, as well as the similarity values and physicochemical properties of all ligands (Additional file 1: Table S2). The overlay of the crystal structure (Additional file 1: Fig. S1) and comparing the amino acid sequence showed that the 22 crystal structure has a high consistency and a complete binding pocket. Due to the Induced Fit Theory, the TβR1 will changes its conformation on binding a ligand. Therefore, there are slight differences in the conformation of 22 co-crystal structures (Fig. 1).

The ligand in 6B8Y (resolution 1.65 Å) with the IC_50_ value of 0.55 nM was the most active inhibitor of 22 molecules in co-crystal structures. Therefore, 6B8Y may be more representative of the active conformation of the target protein. 6B8Y was selected for the evaluation of scoring functions from up to 22 co-crystal structures.

The root-mean-square deviation (RMSD) value (in Å) between the best docking poses of ligands and the conformations in co-crystal structures was selected as the evaluation criterion to check the accuracy of docking program in the reproduction of the binding poses of ligands in crystal structures of TβR1-ligand complexes. Inhibitors in the above-mentioned crystal structures are shown in Fig. 1. The conformations of the inhibitors confirmed by experimental methods. Thus, 22 active conformations of inhibitors in the crystal structure were selected as the reference ligands. These 22 active conformations were re-docked back to the active site of TβR1 by molecular docking. In previous studies, RMSD values within 2.0 Å were acceptable for the molecular docking-based virtual screening [30]. Thus, in this investigation, the docking results with RMSD ≤ 2.0 Å were considered successful, and the docking results with RMSD > 2.0 Å were considered to be failed.

Receiver operating characteristic (ROC) curves were used to distinguish between “active” and “inactive” samples based on the scores. For the evaluation and validation of the sensitivity (SE) and specificity (SP) of scoring functions, a test set covering active and inactive ligands was established by downloading 8958 ligands from the DUD-E database (http://dude.docking.org/). Namely, this test set includes 281 known active ligands and 8677 ligands assumed to be inactive (referred to as decoys). The decoy compounds have similar physicochemical properties but dissimilar 2D topology (Additional file 1: Table S3) [31]. The prepared active or decoy ligands were docked into the binding pocket of TβR1, respectively and the top-scoring pose of each ligand along with its docking score was used for further analysis.

### Molecular docking and scoring functions

Two molecular docking methods, LibDock and CDOCKER in DS software, were performed in this study. LibDock is a docking program developed by Diller and Merz, which uses protein site features referred to as HotSpots [32], while CDOCKER is a grid-based molecular docking method based on CHARMm [33]. Before molecular docking, the ligands in the test set were prepared using the Prepare Ligands protocol to remove duplicates, generate 3D conformations, and remove compounds with undesirable properties. For LibDock, the parameter “Conformation Method” was set to “BEST”, “Max Hits to Save” was set to “1” and “Minimization Algorithm” was set to “Smart Minimizer”. All other parameters were set to default. For CDOCKER, “Top Hits” was set to “1”, “Pose Cluster Radius” was set to ‘0.5’, and others were set to default. Only one top docking pose for each molecule was reported and saved for further analysis. The RMSD values (in Å) between the best docking poses of ligands and the conformations in co-crystal structures were calculated.

The top docking pose for each molecule was re-scored using the Score Ligand Poses protocol in the DS software. The scoring functions, including CDOCKER Scores (CDOCK), LigScore1, LigScore2, PLP1, PLP2, Jain, PMF, PMF04, Ludi Energy Estimate 1 (Ludi 1), Ludi Energy Estimate 2 (Ludi 2), Ludi Energy Estimate 3 (Ludi 3), were used to calculate ligand binding in an active receptor site. The calculated scores were reported as positive values to ensure that higher scores indicated more favorable binding.

### ROC curve analysis

All ligands in the test set (281 active and 8677 inactive) were docked into the binding pocket of TβR1 (PDB ID: 6B8Y). The top docking pose for each ligand was re-scored used the above-mentioned 11 scoring functions. Using a diagnostic trial approach, the SE and SP of each scoring function for distinguishing between “active” and “inactive” samples, were calculated. In the ROC curve, the SE is plotted against 100 − SP for every cut-off point of the variable (score calculated by scoring function). The SE is defined as the ratio of true positive (TP) to the sum of TP and false negative (FN): [TP/(TP + FN)]; and the SP is defined as the ratio of true negative (TN) to the sum of TN and false positive (FP): [TN/(TN + FP)] [14]. The reliability of the scoring function was estimated using the area under the curve (AUC), together with the Chi-square test. A statistically significant difference was defined when P < 0.05. Typically, a ROC curve has an AUC baseline of 0.5, which demonstrates an evenly distributed system. An AUC value closer to 1 indicates good selectivity, whereas a value close to 0.5 indicates random selection.

### Enrichment factor analysis

Taking enrichment factor (EF) as the indicator, the performance of the scoring function as a virtual screening tool applied to TβR1 was evaluated. EF is defined as:

$$ {\text{EF}} = \frac{{\mathop {\text{Hits}}\nolimits_{\text{sampled}}^{X\% } }}{{\mathop N\nolimits_{\text{sampled}}^{X\% } }} \cdot \frac{{\mathop N\nolimits_{\text{total}} }}{{\mathop {\text{Hits}}\nolimits_{\text{total}} }} $$

where $\mathop {\text{Hits}}\nolimits_{\text{sampled}}^{X\% }$ is the number of hit compounds at X% of the database, $\mathop {\text{N}}\nolimits_{\text{sampled}}^{X\% }$ is the number of compounds screened at X% of the database, $\mathop N\nolimits_{\text{total}}$ is the number of compounds in the database, and $\mathop {\text{Hits}}\nolimits_{\text{total}}$ is the number of active compounds in the database. Among the top-rank screened database compounds, the enrichment ability of active compounds is the most noteworthy. Therefore, we mainly focus on the enrichment factor at 0.5%, 1%, and 2% of the ranked database, which are defined as EF^0.5%^, EF^1%^, and EF^2%^.

## Results and discussion

### Assessment of docking methods

Properly docking the ligands to the active site is the most critical step in the virtual screening. The LibDock and CDOCKER docking programs were performed in this study. The results require evaluation to determine the best docking method for the TβR1 protein target and to maximize the probability of success. The performance of the docking programs for predicting the binding mode between ligand and TβR1 was evaluated with a success rate. 22 known active conformations of TβR1 inhibitors were re-docked into the corresponding binding pockets. As virtual screening projects always involve thousands to tens of thousands of ligands, only the top-scoring pose of each ligand was considered as the possible active conformation. The RMSD values between the docked poses with the highest score and those in co-crystal structures are listed in Table 1.

**Table 1:** RMSD values (in Å) between the best docking poses of ligands and the conformations in co-crystal structures for all retrieved actives ligands

NO.	Ligand	MW	RMSD values (Å)	
			LibDock	CDOCKER
1	1RW8	293.338	0.2397	1.5416
2	1PY5	272.304	1.1255	1.1468
3	1VJY	287.319	0.2443	0.3263
4	2WOU	369.418	2.0171	0.3847
5	2WOT	458.509	0.5475	0.2087
6	2X7O	494.627	0.9240	0.6066
7	3GXL	352.392	0.3867	0.4265
8	3FAA	362.36	5.6841	1.5150
9	3HMM	313.356	0.2322	0.1976
10	3KCF	360.409	0.6144	0.2904
11	3TZM	390.435	4.9755	4.8933
12	4X2F	253.259	5.7255	0.4702
13	4X2G	253.259	5.2960	1.1824
14	4X2J	253.259	6.2834	0.3497
15	5E8W	466.531	0.7055	1.2770
16	5E8Z	420.464	8.5303	1.1120
17	5FRI	332.742	4.9761	0.3629
18	5QIK	365.361	0.9489	0.2173
19	5QIL	359.381	0.9670	0.2635
20	5QIM	359.381	6.9569	0.7050
21	5USQ	438.928	1.8278	1.9753
22	6B8Y	374.295	0.2779	0.2861
Success rate			59%	95%

Ligands were named with the PDB ID; MW, molecular weight; docking results with RMSD ≤ 2.0 Å are considered successful, and docking results with RMSD > 2.0 Å are considered to be failed

Results demonstrate that the accuracy of the Lidock program (59%) is much lower than that of the CDOCKER program (95%). Thus, the CDOCKER program was chosen as the docking programs in the subsequent study, based on its reliability.

### Assessment of scoring functions

**Fig. 2:**
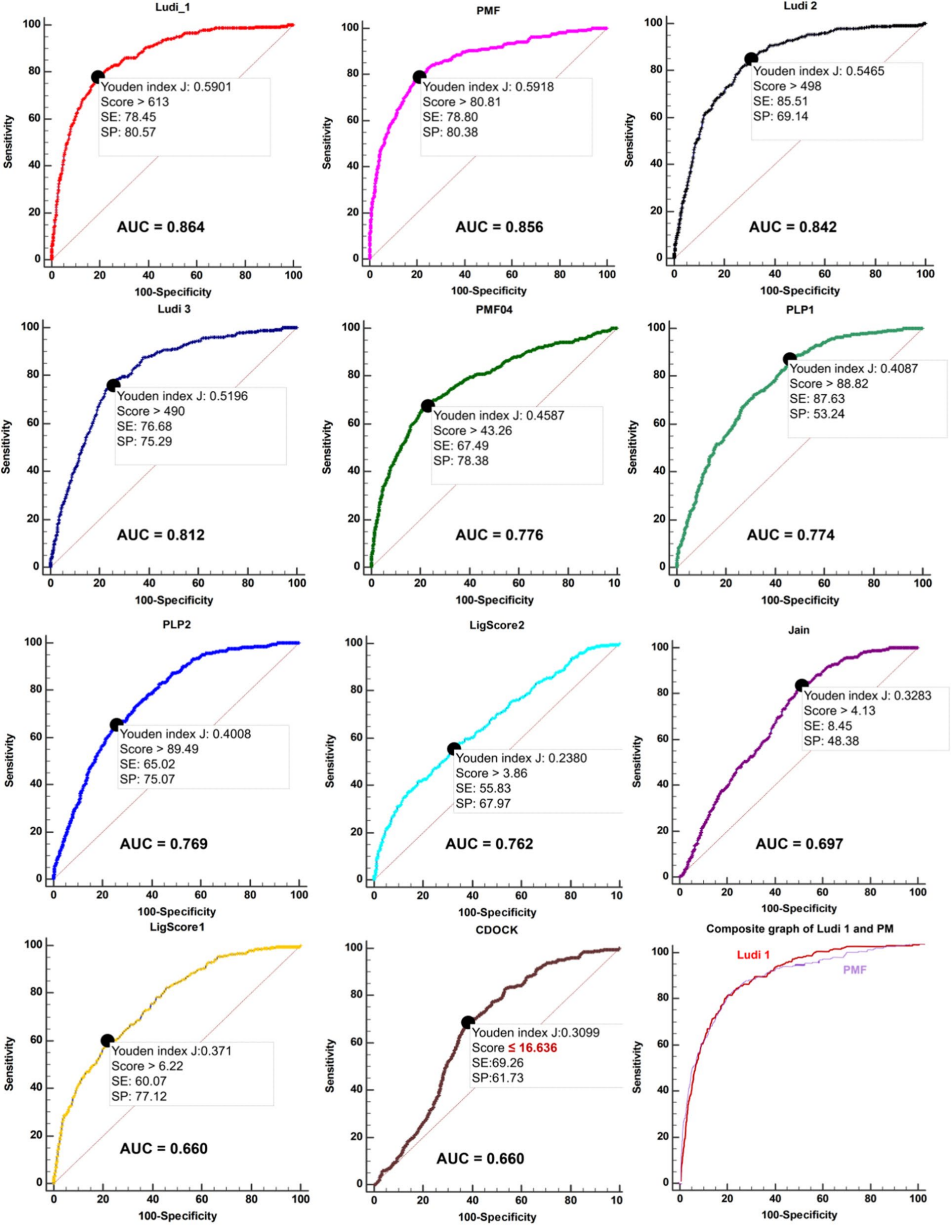
ROC curves for the scoring functions

Scoring and ranking the docked ligands play an important role in virtual screening. The best scoring function for TβR1 should be selected to maximize the chances of success. In this study, 281 known active and 8677 inactive ligands were used to determine the best scoring functions before a larger-scale screening of unknown compounds. In this study, the ROC curves are used to compare the performance of scoring functions in attributing better scores to active than inactive ligands. The docking results showed that, among 8959 ligands, only one active molecule and 22 inactive molecules could not be docked into the active cavity. The best docking poses for each docked ligand were recalculated with 11 scoring functions, respectively. Based on the information of known active and inactive ligands, the ROCs (Fig. 2) were drawn using statistical software, and relevant statistical parameters are listed in Table 2.

**Table 2:** ROC curve results for the scoring functions

Scoring function	AUC			Youden’s index			
	AUC	95% CI	P	Youden index J	Score	SE	SP
Ludi 1	0.864	0.856 to 0.871	< 0.0001	0.5901	> 613	78.45	80.57
PMF	0.856	0.849 to 0.864	< 0.0001	0.5918	> 80.81	78.80	80.38
Ludi 2	0.842	0.834 to 0.849	< 0.0001	0.5465	> 498	85.51	69.14
Ludi 3	0.812	0.803 to 0.820	< 0.0001	0.5196	> 490	76.68	75.29
PMF04	0.776	0.767 to 0.784	< 0.0001	0.4587	> 43.26	67.49	78.38
PLP1	0.774	0.765 to 0.782	< 0.0001	0.4087	> 88.82	87.63	53.24
PLP2	0.769	0.760 to 0.777	< 0.0001	0.4008	> 89.49	65.02	75.07
LigScore2	0.762	0.753 to 0.771	< 0.0001	0.3719	> 6.22	60.07	77.12
Jain	0.697	0.687 to 0.706	< 0.0001	0.3283	> 4.13	84.45	48.38
LigScore1	0.660	0.650 to 0.669	< 0.0001	0.2380	> 3.86	55.83	67.97

*AUC* area under the curve, *95% CI* asymptotic 95% confidence interval, *P* significance level P (area = 0.5), *SE* sensitivity, *SP* specificity

As a higher score indicates a more favorable binding, the result of the CDOCK is unreliable. The ROC curves of Ludi 1, PMF, Ludi 2, Ludi 3, PMF04, PLP1, PLP2, LigScore2, Jain and LigScore1 all tend to the upper left corner, with AUCs of 0.864, 0.856, 0.842, 0.812, 0.776, 0.774, 0.769,0.762, 0.697 and 0.660, respectively (P < 0.0001). All the AUCs of scoring function are significantly larger than those of random distribution (Reference line in Fig. 2, AUC = 0.5), which indicates that the prediction capacity of these 10 scoring functions is better than the random distribution model. The AUC of Ludi 1 is the largest, and the curve is closest to the upper left corner, indicating that Ludi 1 can efficiently distinguish active and inactive molecules. Pairwise comparison of ROC curves shows a significant difference in AUCs between Ludi 1 and other scoring functions (P < 0.0001), except for PMF (P = 0.6170). Given the high accuracy of Ludi 1 and PMF for TβR1, we strongly recommend Ludi 1 and PMF as scoring functions for virtual screening of new inhibitors of TβR1.

Large-scale bioactivity testing is an extremely expensive process. Therefore, it is essential to minimize the number of virtual screening false positives, before a large database search of active molecules and their experimental validation. Therefore, achieving a high positive predictive value (PPV = TP/(TP + FP)) is mandatory. In general, large databases exhibit a low frequency of active ligands. This suggests that increasing SP is more helpful to improve the PPV than altering SE. For example, in this study, active ligands accounted for 3% (280/8958) of the test set. Based on the results form Ludi1, with the increase in SP from 80 to 90%, 95% and 99%, the PPV value increases from 12% (222/1904) to 17% (171/1028), 22% (118/547) and 28% (34/120), respectively. If PMF instead of Ludi 1 as a scoring function, the PPV value increases from 12% (219/1862) to 18% (166/948), 24% (136/569) and 43% (66/151), respectively. As illustrated in Fig. 2, the PMF ROC curve is above the Ludi1 ROC curve in the higher SP region. Furthermore, for the same SP, the PMF ROC curve has higher SE and PPV. Therefore, the PMF scoring function is suitable for this particular research objective.

To obtaining more novel molecular structures for drug design, higher SE must be considered in the virtual screening. When SE = 0.9 (255 active compounds were obtained), a total of 3772 compounds were obtained by PMF, while only 3597 compounds were screened by Ludi1, with fewer false positive ligands. As shown in Fig. 2, the Ludi 1 ROC curve is above the PMF ROC curve in the higher SE region in the upper right corner. For the same SE, the Ludi 1 ROC curve has a higher SP. Therefore, our results indicate that Ludi 1 scoring function has more advantages to this particular objective.

It can be seen from Table 3 that for TβR1, PMF all performs best at EF^0.5%^, EF^1%^, and EF^2%^ among all scoring functions, and Ludi 1 ranks the second. Besides, Jain is the worst among the above 10 scoring functions. In line with ROC curve analysis results, EF evaluation results also support PMF and Ludi 1 as the top two scoring functions.

**Table 3:** EFs of the scoring functions and pharmacophore model A02

Scoring function	EF^0.5%^	EF^1%^	EF^2%^
Ludi 1	11.93	9.58	8.42
PMF	19.38	15.61	12.60
Ludi 2	9.12	7.37	5.80
Ludi 3	7.18	5.68	5.12
PMF04	10.53	7.45	7.81
PLP1	8.61	7.10	4.79
PLP2	7.18	6.03	4.44
LigScore2	5.61	5.61	6.42
Jain	0.72	0.71	1.21
LigScore1	4.31	4.16	5.18
Pharmacophore model A02	12.92	8.16	4.44

Different virtual screening models can be complementary in the search for new inhibitors. In our previous studies, based on the crystal structure of TβR1-BMS22 complex, a pharmacophore model was constructed [14]. In this study, EF^0.5%^, EF^1%^, and EF^2%^ of the pharmacophore model A02 was also evaluated. It can be seen from Table 3 that EFs comparison indicates that the pharmacophore model A02 has very excellent performance at EF^0.5%^, only lower than PMF. Meanwhile, it ranks third at EF^1%^, slightly lower than PMF and Ludi 1. However, the low ranking of the pharmacophore model A02 at EF^2%^ means that the screening results may not be ideal. EF evaluation results show that the pharmacophore model can also obtain excellent virtual screening results in the top-scoring area. Therefore, we recommend that molecular docking and pharmacophore cross-validation can be performed to obtain more accurate virtual screening results.

In conclusion, our results show that PMF and Ludi1 scoring functions have high reliability in the virtual screening of new inhibitors of TβR1. According to the goal of virtual screening, the scoring function can be flexibly selected. To reduce the cost of experimental activity verification, the PMF scoring function should be chosen first. The Ludi 1 scoring function can be chosen first to obtain more novel inhibitors of TβR1.

## Conclusion

Drug target interaction plays an important role in drug discovery based on target [34, 35], and molecular docking can well simulate the interaction between drug and target, which is widely used [36–38]. Molecular docking is routinely used in virtual screening and generally involves two separate steps. The first step is to position ligands into a protein active site correctly, and the second step is scoring and ranking these docked ligands reasonably. An increasingly large number of studies reported that the docking method and the scoring functions are sensitive to receptors.

In this study, we present a thorough investigation of the performance of the LibDock and CDOCKER protocol in DS software on a test set of 22 TβR1 protein–ligand complexes. The results demonstrate that the CDOCKER protocol based on CHARMm is the optimal docking protocol for TβR1. With a successful docking rate of 95%, which means high reliability, the CDOCKER protocol can be efficiently applied for accurate docking in medium-size virtual screening experiments of TβR1.

A dataset including 281 known active and 8677 inactive ligands was used to determine the best scoring function in DS software for scoring ligands posed by molecule docking. The ROC curves are used to compare the performance of scoring functions in attributing better scores to active than inactive ligands. The ROC curves of Ludi 1, PMF, Ludi 2, Ludi 3, PMF04, PLP1, PLP2, LigScore2, Jain and LigScore1 are better than that of the random distribution model, with AUC of 0.864, 0.856, 0.842, 0.812, 0.776, 0.774, 0.769, 0.762, 0.697 and 0.660, respectively. Based on the pairwise comparison of ROC curves, we strongly recommend that Ludi 1 and PMF should be chosen as scoring functions for virtual screening of new TβR1 inhibitors. The PMF ROC curve is above the Ludi 1 ROC curve in the higher SP region in the lower left quarter, thus for the same SP, the PMF ROC curve has higher SE and PPV values. It is advantageous to reduce the number of false positive molecules and the cost of experimental activity verification. Additionally, the Ludi 1 ROC curve is above the PMF ROC curve in the higher SE region in the upper-right corner; therefore, for the same SE, the Ludi 1 ROC curve has higher SP, which is optimal for obtaining more novel molecules.

Consistent with ROC curve, EF evaluation also supports PMF and Ludi-1 as the top two scoring functions. EF evaluation results also demonstrates that the pharmacophore model can get good virtual screening results in the highest score areas. Therefore, molecular docking and pharmacophore cross validation can be used to obtain more accurate virtual screening results.

## Supplementary information

**Additional file 1: Table S1.** Crystal structures of TβR1 with inhibitors reported in PDB. **Table S2.** Physicochemical properties of each crystallographic ligand. MW, molecular weight; HBD, Number of hydrogen bond donors; HBA, Number of hydrogen bond receptors. **Table S3.** Physicochemical properties of compounds in the test set. NC, Number of compounds; MW, molecular weight; nROT, Number of rotatable bonds; nHBA, Number of hydrogen acceptor donors; nHBA, Number of hydrogen bond receptors; nRings, Number of rings. **Fig. S1.** Overlay of 22 TβR1 proteins in the PDB. Except for the Gly367-Gly374 region (far away from the binding site) of 1RW8 and 1PY5, the rest of the crystal structures were well overlapped.

## Data Availability

The datasets used and/or analyzed during the current study are available from the corresponding author on reasonable request.

## Abbreviations

TGF-β: Transforming growth factor-β
TβR1: Transforming growth factor-β type 1 receptor
RMSD: Root-mean-square deviation
CADD: Computer-aided drug design
DBVS: Molecular docking-based virtual screening
ROC: Receiver operating characteristic
SE: Sensitivity
SP: Specificity
TP: True positive
FN: False negative
TN: True negative
FN: False positive
AUC: Area under the curve
95% CI: Asymptotic 95% confidence interval
P: Significance level
EF: Enrichment factor
