# Supplementary material for: Docking-based virtual screening of TβR1 inhibitors: evaluation of pose prediction and scoring functions
Source: BMC Chem. 2020 Aug 14;14(1):52. doi: 10.1186/s13065-020-00704-3 (PMC7427878; doi:10.1186/s13065-020-00704-3)
Supplement: Supplementary file 1 — Additional file 1: Table S1. Crystal structures of TβR1 with inhibitors reported in PDB. Table S2. Physicochemical properties of each crystallographic ligand. MW, molecular weight; HBD, Number of hydrogen bond donors; HBA, Number of hydrogen bond receptors. Table S3. Physicochemical properties of compounds in the test set. NC, Number of compounds; MW, molecular weight; nROT, Number of rotatable bonds; nHBA, Number of hydrogen acceptor donors; nHBA, Number of hydrogen bond receptors; nRings, Number of rings. Fig. S1. Overlay of 22 TβR1 proteins in the PDB. Except for the Gly367-Gly374 region (far away from the binding site) of 1RW8 and 1PY5, the rest of the crystal structures were well overlapped. [file 13065_2020_704_MOESM1_ESM.docx]

**Docking-based virtual screening of TβR1 inhibitors: Evaluation of pose prediction and scoring functions**

Shuai Wang^#^, Jun-Hao Jiang^#^, Ruo-Yu Li and Ping Deng^*^

College of Pharmacy, Chongqing Medical University, Chongqing 400016, China.

^#^These authors contributed equally to this work.

^*^Correspondence: [100865@cqmu.edu.cn](mailto:100865@cqmu.edu.cn).

**Supporting Information List of Contents**

| No. | Content | Page |
| --- | --- | --- |
| 1 | Table S1 Crystal structures of TβR1 with inhibitors reported in PDB | 3-4 |
| 2 | Table S2 Physicochemical properties of each crystallographic ligand. MW, molecular weight; HBD, Number of hydrogen bond donors; HBA, Number of hydrogen bond receptors | 5-11 |
| 3 | Table S3 Physicochemical properties of compounds in the test set. NC, Number of compounds; MW, molecular weight; nROT, Number of rotatable bonds; nHBA, Number of hydrogen acceptor donors; nHBA, Number of hydrogen bond receptors; nRings, Number of rings | 12 |
| 4 | Fig. S1 Overlay of 22 TβR1 proteins in the PDB. Except for the Gly367-Gly374 region (far away from the binding site) of 1RW8 and 1PY5, the rest of the crystal structures were well overlapped | 13 |

**Table S1 Crystal structures of TβR1 with inhibitors reported in PDB**

| PDB | Organism(s) | Released date | Method | Resolution | Literature |
| --- | --- | --- | --- | --- | --- |
| 1RW8 | [Homo sapiens](https://www.rcsb.org/pdb/search/smartSubquery.do?smartSearchSubtype=TreeEntityQuery&t=1&n=9606) | 2005-02-01 | X-RAY | 2.4 Å | Bioorg. Med. Chem. Lett.  **14**: 3581-3584 |
| 1PY5 | [Homo sapiens](https://www.rcsb.org/pdb/search/smartSubquery.do?smartSearchSubtype=TreeEntityQuery&t=1&n=9606) | 2004-07-13 | X-RAY | 2.3 Å | Bioorg. Med. Chem. Lett  **14**: 3581-3584 |
| 1VJY | Homo sapiens | 2004-08-31 | X-RAY | 2 Å | J. Med. Chem.  **47**: 4494-4506 |
| 2WOU | Homo sapiens | 2009-09-22 | X-RAY | 2.3 Å | J. Med. Chem.  **52**: 7901 |
| 2WOT | Homo sapiens | 2009-09-22 | X-RAY | 1.85 Å | J. Med. Chem.  **52**: 7901 |
| 2X7O | Homo sapiens | 2010-10-20 | X-RAY | 3.7 Å | J. Med. Chem.  **53**: 7287 |
| 3GXL | Homo sapiens | 2009-04-21 | X-RAY | 1.8 Å | Bioorg. Med. Chem. Lett.  **19**: 2277-2281 |
| 3HMM | Homo sapiens | 2009-06-23 | X-RAY | 1.7 Å | Bioorg. Med. Chem. Lett.  **19**: 2277-2281 |
| 3FAA | Homo sapiens | 2009-01-27 | X-RAY | 3.35 Å | Bioorg. Med. Chem. Lett.  **19**: 912-916 |
| 3KCF | Homo sapiens | 2009-12-22 | X-RAY | 2.8 Å | Bioorg. Med. Chem. Lett.  **20**: 326-329 |
| 3TZM | Homo sapiens | 2012-05-23 | X-RAY | 1.7 Å | Cell Signal  **24**: 476-483 |
| 4X2F | [Homo sapiens](https://www.rcsb.org/pdb/search/smartSubquery.do?smartSearchSubtype=TreeEntityQuery&t=1&n=9606) | 2015-10-28 | X-RAY | 1.49 Å | J. Med. Chem.  **58**: 457-465 |
| 4X2G | [Homo sapiens](https://www.rcsb.org/pdb/search/smartSubquery.do?smartSearchSubtype=TreeEntityQuery&t=1&n=9606) | 2015-08-12 | X-RAY | 1.51 Å | J. Med. Chem.  **58**: 457-465 |
| 4X2J | [Homo sapiens](https://www.rcsb.org/pdb/search/smartSubquery.do?smartSearchSubtype=TreeEntityQuery&t=1&n=9606) | 2014-12-24 | X-RAY | 1.69 Å | J. Med. Chem.  **58**: 457-465 |
| 5E8W | [Homo sapiens](https://www.rcsb.org/pdb/search/smartSubquery.do?smartSearchSubtype=TreeEntityQuery&t=1&n=9606) | 2016-05-11 | X-RAY | 1.86 Å | Acta Crystallogr. D Struct. Biol.  **72**: 658-674 |
| 5E8Z | [Homo sapiens](https://www.rcsb.org/pdb/search/smartSubquery.do?smartSearchSubtype=TreeEntityQuery&t=1&n=9606) | 2016-05-11 | X-RAY | 1.51 Å | Acta Crystallogr. D Struct. Biol.  **72**: 658-674 |
| 5FRI | [Homo sapiens](https://www.rcsb.org/pdb/search/smartSubquery.do?smartSearchSubtype=TreeEntityQuery&t=1&n=9606) | 2016-07-27 | X-RAY | 2 Å | Med. Chem. Commun.  **7**: 1204-1208 |
| 5QIK | [Homo sapiens](https://www.rcsb.org/pdb/search/smartSubquery.do?smartSearchSubtype=TreeEntityQuery&t=1&n=9606) | 2018-10-31 | X-RAY | 1.58 Å | ACS Med. Chem. Lett.  **9**: 1117-1122 |
| 5QIL | [Homo sapiens](https://www.rcsb.org/pdb/search/smartSubquery.do?smartSearchSubtype=TreeEntityQuery&t=1&n=9606) | 2018-10-31 | X-RAY | 1.98 Å | ACS Med. Chem. Lett.  **9**: 1117-1122 |
| 5QIM | [Homo sapiens](https://www.rcsb.org/pdb/search/smartSubquery.do?smartSearchSubtype=TreeEntityQuery&t=1&n=9606) | 2018-10-31 | X-RAY | 1.75 Å | ACS Med. Chem. Lett.  **9**: 1117-1122 |
| 5USQ | [Homo sapiens](https://www.rcsb.org/pdb/search/smartSubquery.do?smartSearchSubtype=TreeEntityQuery&t=1&n=9606) | 2017-04-12 | X-RAY | 2.55 Å | Bioorg. Med. Chem. Lett.  **27**: 1955-1961 |
| 6B8Y | [Homo sapiens](https://www.rcsb.org/pdb/search/smartSubquery.do?smartSearchSubtype=TreeEntityQuery&t=1&n=9606) | 2018-02-07 | X-RAY | 1.65 Å | Bioorg. Med. Chem.  **26**: 1026-1034 |

**Table S2 Physicochemical properties of each crystallographic ligand. MW, molecular weight; HBD, Number of hydrogen bond donors; HBA, Number of hydrogen bond receptors**

| PDB | Ligand | Active data | ALogP | MW | HBD | HBA |
| --- | --- | --- | --- | --- | --- | --- |
| 1RW8 | 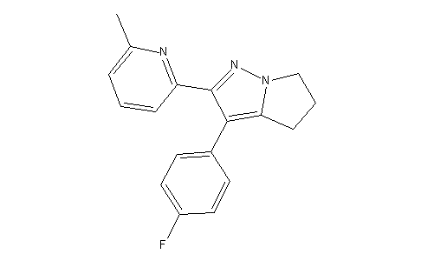  3-(4-fluorophenyl)-2-(6-methylpyridin-2-yl)-5,6-dihydro-4h-pyrrolo[1,2-b]pyrazole | IC_50_=15 nM | 4.112 | 293.338 | 0 | 2 |
| 1PY5 | 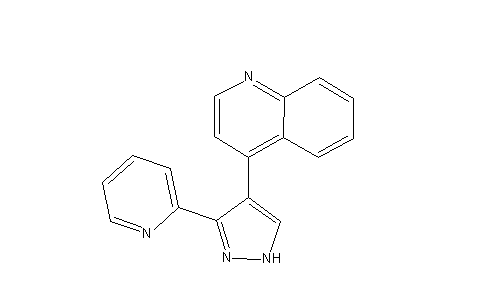 4-(3-pyridin-2-yl-1h-pyrazol-4-yl)quinoline | IC_50_=59 nM | 3.066 | 272.304 | 1 | 3 |
| 1VJY | 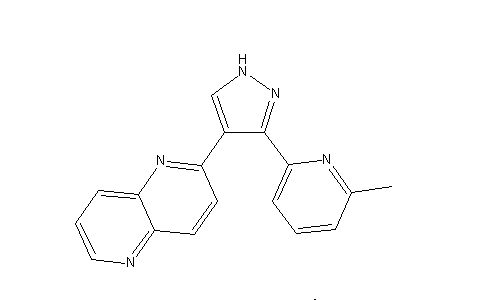 2-[5-(6-methylpyridin-2-yl)-2,3-dihydro-1H-pyrazol-4-yl]-1,5-naphthyridine | IC_50_=23 nM | 3.055 | 287.319 | 1 | 4 |
| 2WOU | 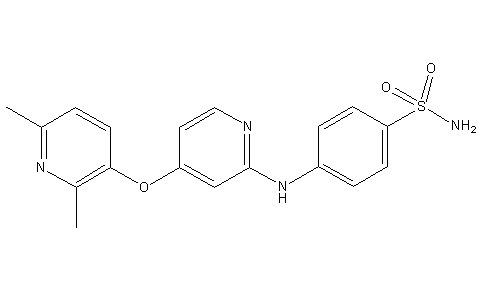 4-({4-[(2,6-dimethylpyridin-3-yl)oxy]pyridin-2-yl}amino)benzenesulfonamide | IC_50_=72 nM | 1.942 | 369.418 | 2 | 7 |
| 2WOT | 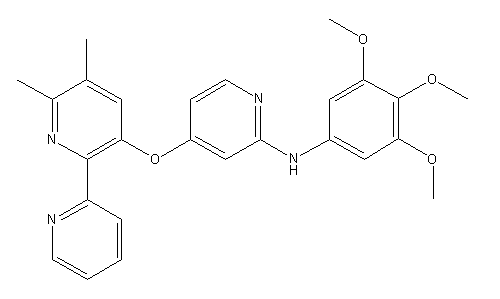 4-({4-[(2,6-dimethylpyridin-3-yl)oxy]pyridin-2-yl}amino)benzenesulfonamide | IC_50_=44 nM | 5.122 | 458.509 | 1 | 8 |
| 2X7O | 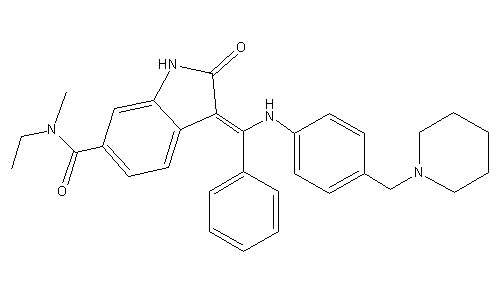 (3Z)-N-ethyl-N-methyl-2-oxo-3-(phenyl{[4-(piperidin-1-ylmethyl)phenyl]amino}methylidene)-2,3-dihydro-1H-indole-6-carboxamide | IC_50_=34 nM | 4.574 | 494.627 | 2 | 4 |
| 3GXL | 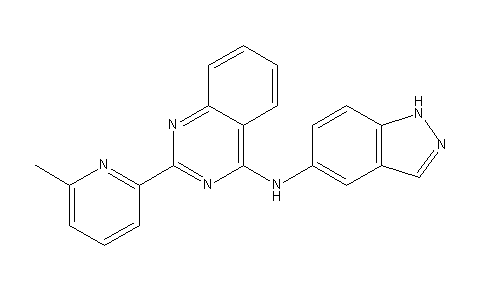 N-1H-indazol-5-yl-2-(6-methylpyridin-2-yl)quinazolin-4-amine (GW857175) | IC_50_=25 nM | 4.301 | 352.392 | 2 | 5 |
| 3HMM | 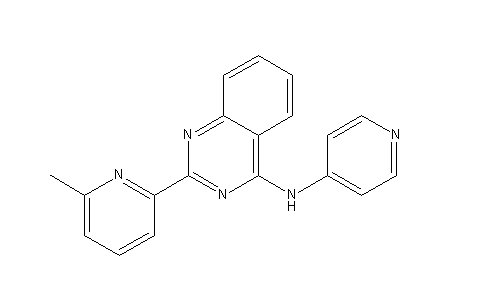 2-(6-methylpyridin-2-yl)-N-pyridin-4-ylquinazolin-4-amine (GW855857) | IC_50_=25 nM | 3.507 | 313.356 | 1 | 5 |
| 3FAA | 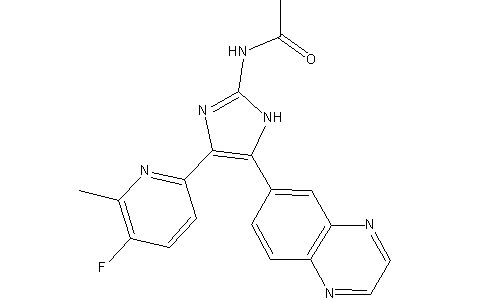 N-[4-(5-fluoro-6-methylpyridin-2-yl)-5-quinoxalin-6-yl-1H-imidazol-2-yl]acetamide | Ki=7.1 nM | 2.366 | 362.36 | 2 | 5 |
| 3KCF | 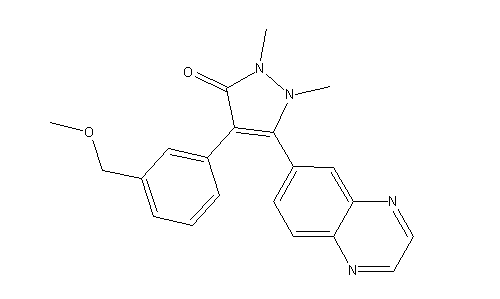 4-[3-(methoxymethyl)phenyl]-1,2-dimethyl-5-quinoxalin-6-yl-1,2-dihydro-3H-pyrazol-3-one | Ki=35 nM | 1.623 | 360.409 | 0 | 5 |
| 3TZM | 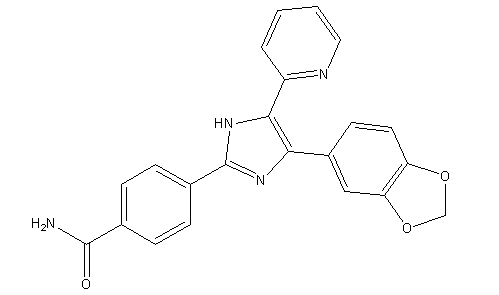 4-[5-(1,3-benzodioxol-5-yl)-4-(pyridin-2-yl)-1H-imidazol-2-yl]benzamide (SB431542) | IC_50_=94 nM | 2.555 | 390.435 | 2 | 5 |
| 4X2F | 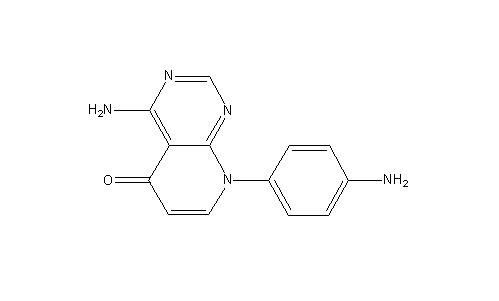 4-amino-8-(4-aminophenyl)pyrido[2,3-d]pyrimidin-5(8H)-one | - | 0.966 | 253.259 | 2 | 6 |
| 4X2G | 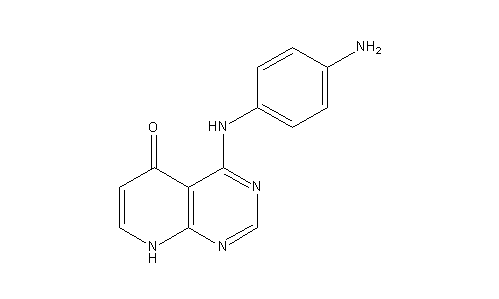 4-[(4-aminophenyl)amino]pyrido[2,3-d]pyrimidin-5(6H)-one | - | 1.316 | 253.259 | 3 | 6 |
| 4X2J | 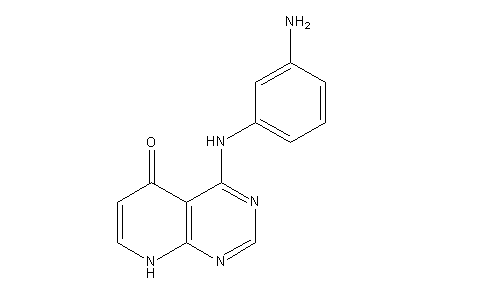 4-[(3-aminophenyl)amino]pyrido[2,3-d]pyrimidin-5(8H)-one | - | 1.316 | 253.259 | 3 | 6 |
| 5E8W | 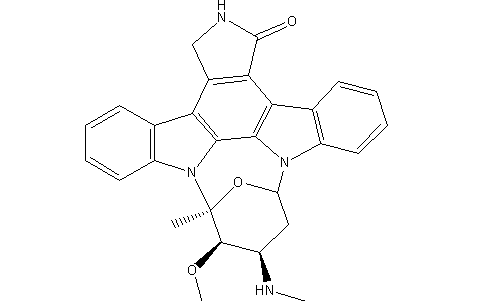 STAUROSPORINE | - | 3.819 | 466.531 | 2 | 4 |
| 5E8Z | 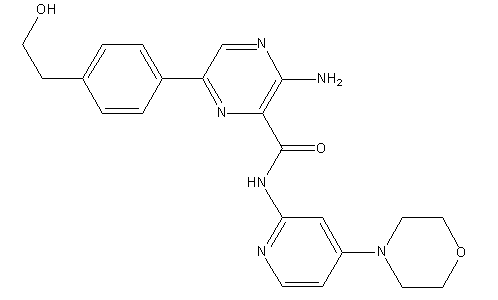 3-amino-6-[4-(2-hydroxyethyl)phenyl]-N-[4-(morpholin-4-yl)pyridin-3-yl]pyrazine-2-carboxamide | - | 0.897 | 420.464 | 3 | 9 |
| 5FRI | 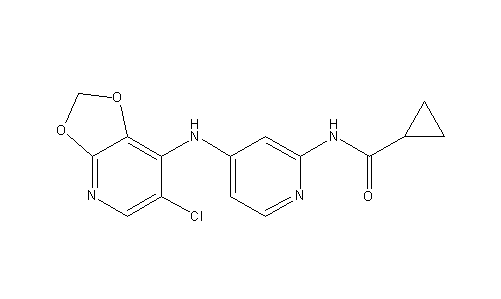 N-[4-[(6-chloro-[1,3]dioxolo[4,5-b]pyridin-7-yl)amino]-2-pyridyl]cyclopropanecarboxamide | IC_50_=4 nM | 2.467 | 332.742 | 2 | 6 |
| 5QIK | 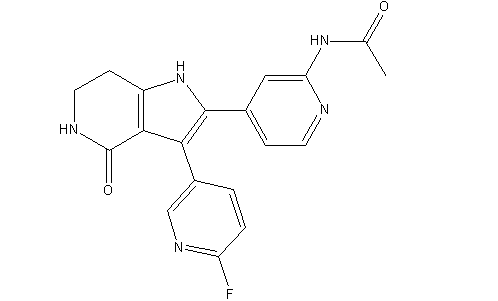 N-{4-[3-(6-fluoropyridin-3-yl)-4-oxo-4,5,6,7-tetrahydro-1H-pyrrolo[3,2-c]pyridin-2-yl]pyridin-2-yl}acetamide | IC_50_=22 nM | 1.484 | 365.361 | 3 | 4 |
| 5QIL | 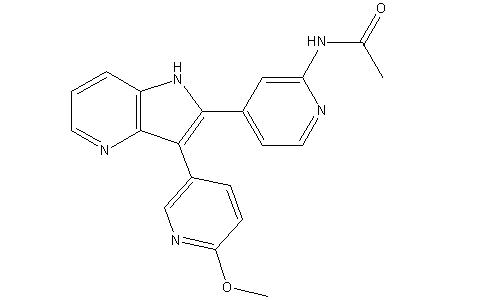 N-{4-[3-(6-methoxypyridin-3-yl)-1H-pyrrolo[3,2-b]pyridin-2-yl]pyridin-2-yl}acetamide | IC_50_=3 nM | 2.61 | 359.381 | 2 | 5 |
| 5QIM | 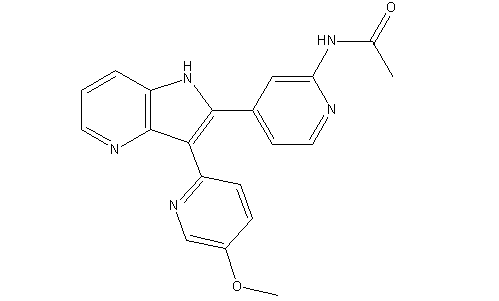 N-{4-[3-(5-methoxypyridin-2-yl)-1H-pyrrolo[3,2-b]pyridin-2-yl]pyridin-2-yl}acetamide | IC_50_=6 nM | 2.499 | 359.381 | 2 | 5 |
| 5USQ | 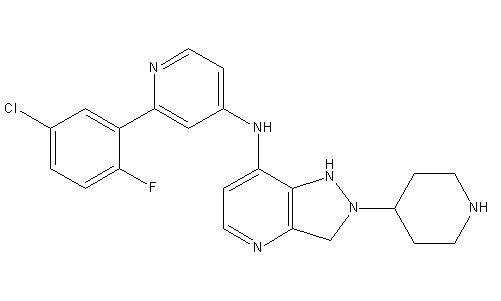 N-[2-(5-chloro-2-fluorophenyl)pyridin-4-yl]-2-[(piperidin-4-yl)methyl]-2H-pyrazolo[4,3-b]pyridin-7-amine | - | 3.704 | 438.928 | 3 | 6 |
| 6B8Y | 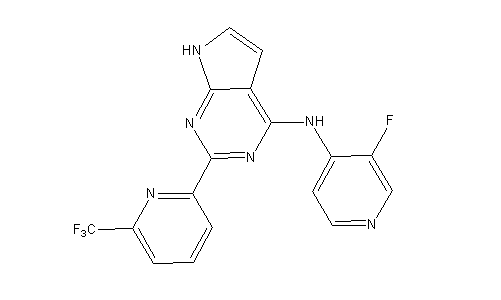 N-(3-fluoropyridin-4-yl)-2-[6-(trifluoromethyl)pyridin-2-yl]-7H-pyrrolo[2,3-d]pyrimidin-4-amine | IC_50_=0.55 nM | 3.956 | 374.295 | 2 | 5 |

*The activity (IC50 or Ki) of compounds in 4X2F, 4X2G, 4X2J, 5E8W, 5E8Z and 5USQ was not reported in the original literature.

**Table S3 Physicochemical properties of compounds in the test set. NC, Number of compounds; MW, molecular weight; nROT, Number of rotatable bonds; nHBA, Number of hydrogen acceptor donors; nHBA, Number of hydrogen bond receptors; nRings, Number of rings**

|  | NC | MW | nROT | nHBD | nHBA | nRings |
| --- | --- | --- | --- | --- | --- | --- |
| Active | 281 | 251-565 | 2-8 | 0-5 | 2-8 | 3-6 |
| Inactive | 8677 | 231-556 | 0-10 | 0-6 | 1-9 | 1-10 |


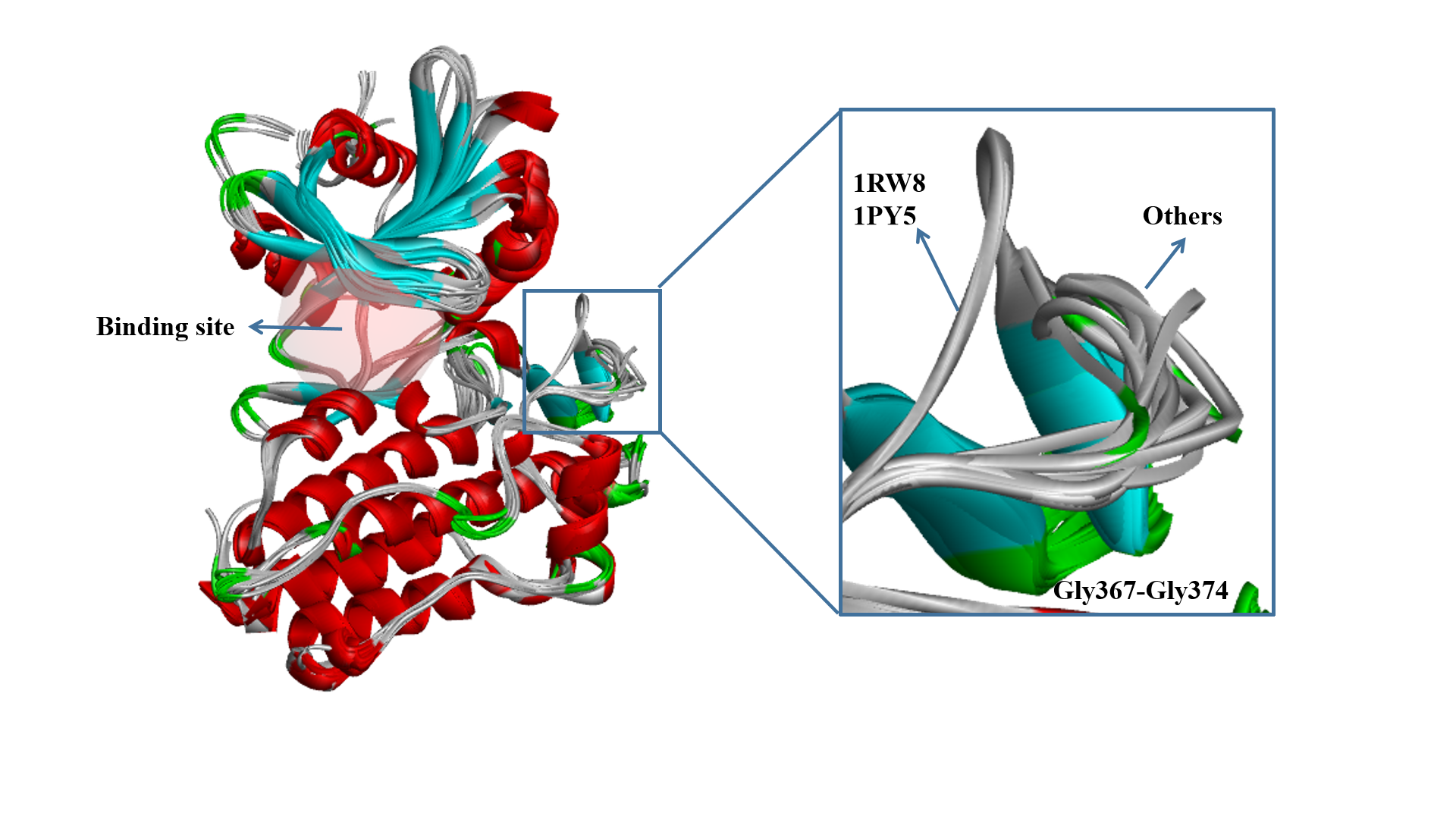


**Fig. S1** Overlay of 22 TβR1 proteins in the PDB. Except for the Gly367-Gly374 region (far away from the binding site) of 1RW8 and 1PY5, the rest of the crystal structures were well overlapped
